# Potential application of aminiotic stem cells in veterinary medicine

**DOI:** 10.21451/1984-3143-AR2018-00124

**Published:** 2020-05-22

**Authors:** Carlos Eduardo Ambrósio, Jéssica Rodrigues Orlandin, Vanessa Cristina Oliveira, Lina Castelo Branco Motta, Priscilla Avelino Ferreira Pinto, Vitória Mattos Pereira, Letícia Ribeiro Padoveze, Rafael Garcia Karam, Alessandra de Oliveira Pinheiro

**Affiliations:** Department of Veterinary Medicine, Faculty of Animal Science and Food Engineering, University of São Paulo, Pirassununga, São Paulo, Brazil.

**Keywords:** amniotic membrane, stem cells, therapy

## Abstract

In regenerative medicine stem cell biology has become one of the most interesting and more often studied subject. The amniotic membrane is the innermost layer of the fetal membranes and is considered a potential tool to treat many pathologies. It is used because it can be collected from discarded fetal material and is a rich source of stem cells with high proliferation and plasticity ratio capable of proliferating and differentiate *in vitro*. We propose to elucidate the characteristics and potencial clinical application of cells derived of amniotic membrane in veterinary medicine.

## Introduction

Stem cells have a capacity for differentiation and self-renewal. The great interest in science by these cells occur due to its immunomodulatory factors and tissue repair that make possible the use in therapies and innovative treatments (Faita *et al*., 2016). 

Several adult tissue are source of stem cells with therapeutic potential, such a adipose tissue (Zuk *et al*., 2002; Rada *et al*., 2011), bone marrow (Kastrinaki *et al*., 2008) and peripheral blood (Villaron *et al*., 2004). There is possible to use the fetal membranes as source too, such as amniotic membrane (AMSC) (Park *et al*., 2012), amniotic fluid (Perin *et al*., 2007; Steigma and Fauza, 2007; You *et al*., 2008) and placenta (Steigman an Fauza, 2007; Barlow *et al*., 2008).

The fetal membranes are essential for embryonic development, as they provide maternofetal exchange. It consists of four different membranes: amnion, chorion, the vitelline sac and the allantoic sac (Mossman, 1987). The amnion is the innermost layer of fetal membranes, and contains a thick basement membrane and an avascular stroma (Malak and Bell, 1994) it acts as an additional maternofetal barrier (Leiser and Kaufmann, 1994). Other potential studies involved embryo development and stem cell niches is also highlighted in our data in canines (Martins *et al*., 2011; Pieri *et al*., 2015; De Souza *et al*. 2018).

Amniotic membrane stem cells (AMSC) have previously been described for their immunoregulatory properties (Bossolasco *et al*., 2006), their differentiate and self-renew potential (Kita *et al*., 2010) and vigorous cell proliferation. The AMSC are isolated from different species as canine, feline, equine, suine, murine and humans. The application of AMSC occur especially in dogs (Park *et al*., 2012; Cardoso *et al*., 2016) and the cats (Vidane *et al*., 2017), although the murine and suine are good specie models for humans studies. 

Thus, this study aimed to carry out amniotic stem cells review about their applicability in the treatment in veterinary medicine.

## Stem cells lineages

Discovered by Becker *et al*. (1963), the stem cells are undifferentiated cells able to perform self-renewal and differentiate into the most diverse functional cell types (Park *et al*., 2015; Dai *et al*., 2016; Sobhani *et al*., 2017), thus being considered promising sources for the use in tissue engineering and organ regeneration.

Stem cells can be classified according to their potential for differentiation. Totipotent cells are those able to differentiate in all cell lines, including extra-embryonic tissues, examples of totipotent cells include the zygote and its early cleavage products. The pluripotent cells are those capable of differentiating only in cells belonging to the three germ layers (Morgani *et al*., 2013) as embryonic stem cells (ESCs) (Slack, 2018) and induced pluripotent stem cells (iPSCs), somatic cells reprogramed do pluripotency (Takahashi and Yamanaka 2006). The iPSCs were initially produced of cells of mice and humans, however, there is currently reprogramming cells several animal species (Takahashi and Yamanaka, 2006; Takahsshi *et al*., 2007; Zhang *et al*., 2015; Gonçalves *et al*., 2017). Thus, besides the great importance for studies with a therapeutic objective, the IPSCs are also important in veterinary medicine for the maintenance of genetic material of animals with great economic importance (Kumar *et al*., 2015). 

Another type of classification are the multipotent cells, which have ability to differentiate into all cell types within a given lineage (Ratajczak *et al*., 2014; Khanlarkhani *et al*, 2016). Although multipotent cells have a lower potential for differentiation, they are considered an important source for cellular therapy (Mirzaei *et al*., 2018), since they have genetic stability, low immunological profile and slower metabolism when compared to pluripotent stem cells, characteristics that decrease the risk of rejection (Liao and Tse, 2013). A further advantage of multipotent stem cells is the accessibility, and the fact that they can be isolated from the most diverse cell types (Khanlarkhani *et al*., 2016; Mirzaei *et al*., 2018). 

The mesenchymal stem cell (MSC) is a type of multipotent stem cell present in most adult tissues, a heterogeneous cell that plays a key role in the development and renew of organs (Klein, 2016). Several reports have shown that MSCs are commonly isolated and characterized from adipose tissue, bone marrow, umbilical cord, liver, as well a fetal attachments such as yolk sac and amnion (Seo *et al*., 2009; Uranio *et al*., 2011; Wenceslau *et al*., 2011; Reich *et al*., 2012; Mançanares *et al*., 2015). This adaptability and easy access make this stem cell a great candidate for use in transplants and therapies.

## Mesenchymal stem cells

The MSCs were described by Friedenstein *et al*. (1970) in bone marrow as a component of the marrow stromal cell population that collectively supports hematopoietic stem cell renew and differentiation (Largeault, 2004; Martins *et al*., 2007). There are MSC niches in many adult tissues and organs. These cells play an active role in the homeostasis of these sites and can be isolated from the umbilical cord blood and matrix, adipose tissue, synovial membranes and embryonic and extraembryonic tissues.

According to the International Society of Cell Therapy the criteria for the characterization of human MSC are adherence to the plastic, positive expression for the surface markers CD105, CD73 and CD90, negative expression for the markers CD45, CD34, CD14 or CD11b, CD79a or CD19 and the HLA-DR surface molecules, capability to differentiate (osteoblasts, adipocytes and chondrocytes) *in vitro* (Dominici *et al*., 2006). According to Casteilla *et al*. (2011), Tharasanit *et al*. 2011), Mançanares *et al*. (2015), MSCs exhibit fibroblastoid morphology in culture.

MSC are easy to isolate, cultivate, and manipulate. These cells have immunoregulation and immunosuppressive characteristics, great plasticity and the potential for therapeutic applications for a variety of clinical conditions (Nardi and Meirelles, 2006; Oliveira *et al*., 2014; Samsoraj *et al*., 2017). 

## Amniotic membrane

The amnion is the most internal extra-embrionary membrane, composed of a thin, elastic, translucent and semi-permeable membrane, derived from the ectoderm that covers the fetus and is closely connected to the chorionic membrane (Fernandes *et al*., 2012; Favaron *et al*., 2015).

Amniotic membrane (AM) is avascular tissue (Miglino *et al*., 2006; Dua *et al*., 2004). It has the function of involving the embryo and delimits the amniotic cavity, which is filled by amniotic liquid to avoid mechanic shocks (Moore and Persaud, 2008; Mamede *et al*., 2012; Koob *et al*., 2014).

Basically, the amniotic membrane is composed of two cell types. The epithelial layer composed by the epithelial amniotic cells which present a cuboidal/columnar morphology derived from ectoderm. And the second cell type is composed of mesenchymal stromal cells that are derived from mesoderm. Both populations have similar markers and have the potential to differentiate *in vitro* in the main mesodermal lineages (Chang *et al*., 2010; Díaz-Prado *et al*., 2011).

The AM has particular characteristics such as anti-inflammatory, anti-bacterial, anti-viral and immunological action. Also contains numerous growth factors, cytokines, and signaling molecules that play important roles in fetal development and gestation (Kogan *et al*., 2018).

Due to the multipotent properties, cells from the amniotic membrane have been investigated for being an attractive source for tissue transplantation (Mamede *et al*., 2012).

## Amniotic Membrane Stem Cells (AMSC)

The AMSC are derived from extraembryonic mesoderm and are randomly distributed in the extracellular matrix of amnio which are rich in collagen and laminin fibers. They are multipotent cells with characteristics that favor their use, such as low immunogenicity and carcinogenicity, production of regulatory molecules of the immune system, high plasticity and propagation in culture and presence of high concentration of lysosomes (Chang *et al*., 2010; Cremonesi *et al*., 2011; Vidane *et al*., 2014; Cardoso *et al*., 2016; Faita *et al*., 2016; Magatti *et al*., 2016; Miki *et al*., 2016).

The AMSC demonstrates absent immunogenic properties and production of anti-inflammatory and bactericidal substances that provide their use in the treatment of skin wounds, cutaneous ulcers, and ophthalmic disorders such as corneal ulcers (Díaz-Prado *et al*., 2011; Parolini *et al*., 2008). 

Furthermore, the AMSC are considered excellent sources of stem cells for regenerative therapies of the nervous system due to neural differentiation ability (Uranio *et al*., 2011; Park *et al*., 2012). 

These cells have advantages over adult stem cells because they preserve embryonic, immunosuppressive and pluripotent characteristics, which are confirmed by the expression of embryonic markers such as OCT-4 and NANOG (Cremonesi *et al*., 2011; Vita *et al*., 2012; Saulnier *et al*., 2016). Other advantage of AMSC is because they are easy to acquire, offer no damage to donors, and have a lower immune response, making them important in research for regenerative medicine, since inflammation and immunogenicity are crucial factors for successful transplantation (Kim *et al*. 2014). 

**Figure 1:**
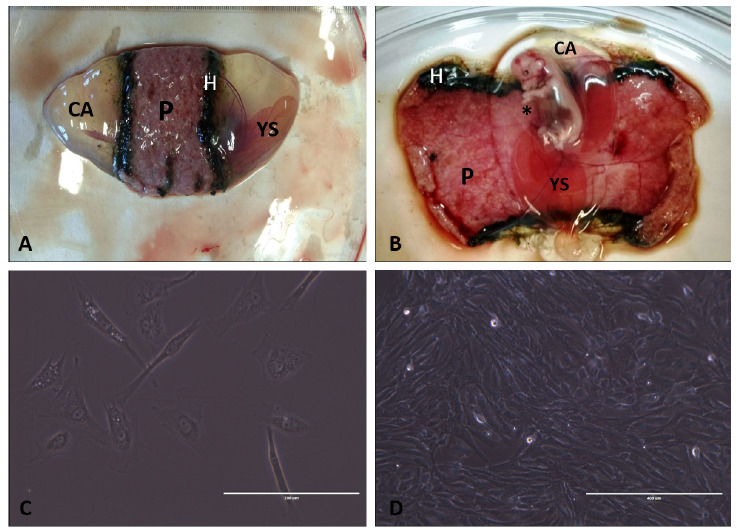
A and B: Canine fetus developing, with possible visualization of placental (P) structures, marginal hematoma (H), chorioallantoic membrane (CA), yolk sac (YS) and amniotic membrane (*). C and D: photomicrographs of AMCs in primary culture on different days of culture. C, with 4 days and D with 10 days.

The cells derived from the amniotic membrane have the same characteristics of mesenchymal stem cells, according to the International Society of Cell Therapy criteria (Cardoso *et al*., 2016), and their morphology shows fibroblast characteristics (Fig. 1).

## Isolation Protocols for AMSC

There are many types of isolation protocols for AMSC in each species, although they are quite similar. In this review, we will describe the protocols most use with different species in our lab.

For species which the site of collection is more contaminated, as swine and horses, we strongly suggest the refrigeration of samples (1h - 2h) before the beginning of the mechanic process, in order to reduce the bacterial activity. So, the amniotic membrane must be mechanically dissect and isolated from embryonic attachments. Under sterile conditions, the tissue must be washed with PBS supplemented with 1% penicillin/streptomycin (P/S, Sigma, USA). After this step, the sample will be transfer to a clean dish and mince with scalpels to become homogeneous and pasty.

After the above-described process, the sample is submitted to chemical digestion using collagenase type I (Sigma, USA) which the concentration and time are described below to each species (Tab. 1).

After digestion the collagenase enzyme has to be neutralizing using the same volume of expansion media to the cell suspension. So, the digested sample is centrifuged at 1600 rpm/5 min, and then cell pellet is obtained to be culture with the expansion media and keep at 37°C and 5% CO2.

**Table 1:** Culture protocols of AMSC from different species.

Animal	% collagenase	Time	Expanded Medium	Reference
Canine	1-2 mg/mL	3 - 4 h	high-glucose Dulbecco’s modified Eagle’s medium (HG- DMEM) + 10% SFB + 1% P/S + 2 mM L-glutamine	Park *et al*., 2012
Feline	1 mg/mL	3 h	HG-DMEM + 10% SFB + 1% P/S + 2 mM L-glutamine	Vidane *et al*., 2017
Equine	0,93 mg/mL + 20 mg/mL DNAse	3 h	HG-DMEM + 10% SFB + 10 ng/ml epidermal growth factor (EGF; Sigma) + 1% P/S + 0.25 µg/ml amphotericin B + 2 mM L-glutamine	Lange-Consiglio, *et al*., 2013a
Swine	0,93 mg/mL + 20 mg/mL DNAse	3 h	HG-DMEM +10% SFB + 10 ng/mL EGF + 1% P/S + 0.25 µg/ml amphotericin B + 2 mM L-glutamine	Lange-Consiglio *et al*., 2015
Human	25 mg/mL	1h	HG-DMEM + 10% SFB + 1% P/S	Dizaji *et al*., 2017
				

## Applications and clinical implications of amniotic stem cells in Veterinary Medicine: a pet market model

### Neurologic applications

The AMSC is considered a source of election to treat spinal cord injury. Sankar *et al*. (2003), showed good interaction of human amniotic membrane stem cells when applied in *iatrogenic spinal cord injury* in monkeys, and they observed a significant remyelination, as well as their ability to modulate the glial scar. And a similar study was performed by Zhi-Yuan *et al*. (2006) who observed a returning of motor function in the hind limbs of treated animals. Furthermore, Meng *et al*. (2008) studied the co-transplantation of AMSC with stem cells of neuronal origin in rats with *chronic spinal cord injury*. And again, the results demonstrated a significant locomotor improvement in addition to neuronal survival and differentiation.

Kakishita *et al*. (2003) verified that human amniotic epithelial cells secrete biologically active neurotrophins and can enhance the survival of dopamine neurons, being a promising therapeutic tool in Parkinson's disease. As well as Yang *et al*. (2009) who also observed a increasing of dopamine and its metabolic products in the striatum in rats with Parkinson’s disease.

### Ischemic dysfunction

Kim and Choi (2011) observed neovascularization in mouses suffering of limb ischemia. They administered intramuscularly hAMSCs on the leg whose femoral vessels were ligated. The blood flow recovery was significantly higher in the transplanted group, when compared with the control group, being an attractive source for the treatment of ischemic diseases.

And Tao *et al*. (2012) transplanted human amniotic membrane stem cells transfected with the brain derived neurotrophic factor (BDNF) into the brains of rats with induced stroke. This technique ameliorated the behavioral dysfunction and reduced the infarct volume, improving functional recovery.

### Musculoskeletal disorders

The stem cell therapy is a routine treatment in horses, and the amnion plays an important role in this scenario. In mares with endometriosis, the AMSC cells were used because of their potential to improve cell replenishment based on gene expression profiling when low proliferation of uterine cells is associated to pregnancy failure in an *in vivo* study, showing lower rate of reinjury and faster resume to their activities (Corradetti *et al*., 2014). In tendon repair, the re-injury was lower in treatment with AMSC than compared with BM-MSC ascertained by ultrasonography (Lange-Consiglio *et al*., 2013b). Still, biomaterials composed for amniotic membrane has attracted attention. They have being associated to better mechanical properties in tendons injuries, plus this membrane has been used to accelerate healing wounds (Violini *et al*., 2012; Hortensius *et al*., 2016).

### Others applications

A promising use of large animal models must be done before clinical trial and confirm efficacy of therapy and controversial results must be followed (Gonçalves *et al*, 2014). Kamiya *et al*. (2005) showed efficacy in suppressing corneal inflammatory reactions, when topically applied human amniotic membrane culture supernatant in mices with induced corneal neovascularization.

Vidane *et al*. (2017) evaluated the effects of intravenous administration of allogeneic feline amniotic membrane stem cells in cats with naturally occurring chronic kidney disease. Despite the kidney architecture and morphology did not change during the treatment, the tranplantation showed renoprotective effect, improved renal function, delaying the progression of the disease and stabilizing the clinical condition of the animals.

And finally, the AMSC are described into application in humans to aid in treatment of cutaneous wounds, burns, and superficial ocular reconstruction (Ward and Bennett, 1984; Avila *et al*., 2001; Gomes *et al*., 2005).
